# The effectiveness of weighted least squares means and variance adjusted based fit indices in assessing local dependence of the rasch model: Comparison with principal component analysis of residuals

**DOI:** 10.1371/journal.pone.0271992

**Published:** 2022-09-15

**Authors:** HyunSuk Han

**Affiliations:** Kyungil University, Gyeongsan, Gyeongbuk, Republic of Korea; UNITED STATES

## Abstract

Local independence is a principal assumption of applying latent variable models. Violations of this assumption might be stemmed from dimensionality (trait dependence) and statistical independence of item responses (response dependence). The purpose of this study is to evaluate the sensitivity of weighted least squares means and variance adjusted (WLSMV) based global fit indices to violations of local independence in Rasch models, and compare those indices to principal component analysis of residuals (PCAR) that is widely used for Rasch models. Dichotomous Rasch model is considered in this simulation study. The results show that WLSMV-based fit indices could detect trait dependence, but are to be limited with regard to response dependence. Additionally, WLSMV-based fit indices have advantages over the use of PCAR since WLSMV-based global fit indices are consistent regardless of sample size and test length. Though it is not recommended to apply exact benchmarks for those indices, they would provide practitioners with a method for evaluating the degree to which assumption violation is problematic for their data diagnostic purpose.

## Introduction

Given the relationships of item response theory (IRT) models to confirmatory factor analysis (CFA) models, IRT model misspecifications might be detectable through model fit indices commonly used in categorical CFA. IRT models share many features with CFA models, and in some cases are equivalent to CFA models [1]. Hence, IRT model misspecifications may be detectable through model fit indices commonly used in CFA. The Rasch model is mathematically equivalent to one-parameter IRT models [2], and it has been widely used to estimate item and ability parameters from measurement data. From the perspective of CFA, a unidimensional Rasch model can be considered as equivalent to a particular form of a unidimensional, categorical CFA model. Specifically, if the loadings of the unidimensional, categorical CFA model are constrained to be equal and fixed to one, the statistical expression of the model is equivalent to the unidimensional Rasch model [3].

Previous research [4] showed that weighted least squares means and variance adjusted (WLSMV) global model fit indices used in structural equating modeling practice are sensitive to person parameter estimate RMSE and item difficulty parameter estimate RMSE that results from local dependence in two-parameter logistic (2-PL) IRT models, particularly when conditioning on number of test items and sample size. In this study, I hypothesize that there is a clear relationship between WLSMV-based fit indices and LD-induced inaccurate parameter estimate when fitting models in which the discrimination parameter is fixed. That is, Rasch model applications will demonstrate a clearer relationship between these fit indices and inaccurate parameter estimate from LD than was found for 2-PL models in Huggins-Manley and Han [4]. If this hypothesis is supported by the study, practitioners can use the fit indices that would be useful supplementary statistics to detect problematic LD in Rasch model applications. This would be a benefit to the measurement field as many current methods for detecting LD require complex computations, provide information at the item or item-pair level, and/or provide vague information with respect to how problematic the LD is for obtaining unbiased parameter estimates [5–13]. Therefore, the purpose of this study is to evaluate the sensitivity of WLSMV-based global fit indices to violations of local independence in Rasch models, particularly violations that result in inaccurate person estimate and/or item parameter estimate. Additionally, to examine whether those fit indices are related to other widely used methodologies in applied Rasch modeling, the author compares the results to principal component on residual (PCAR) analysis. PCAR is the most popular method particularly in assessing local independence assumption in Rasch models. The use of the first eigenvalue from PCAR is used to check local dependency due to the presence of multidimemtionality [14, 15]. Several rules of thumb regarding the first eigenvalue from PCAR were suggested [16–18]. Since the use of PCAR for local independence testing serves as a goodness of fit statistic as WLSMV-based fit indices does in this study, PCAR results are provided in the comparison of WLSMV-based fit indices. The Rasch model for binary responses is considered in this study. The specific research questions are:

1. Are CFI, TLI, and RMSEA indices from WLSMV estimation of binary Rasch models sensitive to LD that causes inaccurate estimates of item and person parameters?
2. Is the answer to Question 1 dependent on the number of observed variables, sample size, type of LD (trait or response dependence), and/or magnitude of LD?
3. Are the WLSMV-based fit indices useful in the comparison of PCAR results?

### Types of local dependence in rasch model

Marais and Andrich [19] distinguished two types of violations of LD. One, a violation of unidimensionality, is called trait dependence (TD). The other, a violation of statistical independence, is called response dependence (RD).

#### Trait dependence

Marais and Andrich [19] pointed out that the TD can be considered a violation of unidimensionality. It is common that the scale used in social sciences tends to be constructed as a set of different subsets even though the instrument is intended to measure a single psychological trait. The instrument designed as a composition of the subsets has the advantage of obtaining measurement validity because the subsets design is capable of reaching out the various aspects of the trait. However, it has risk of violating the unidimensionality. Thus, if the subsets in the instrument measure a different latent trait, the instrument can no longer be considered a single instrument to measure a single latent trait.

In this study, under the Rasch model, TD can be formularized as

$$P\left(X_{ip}^{\left(s\right)}=1|\theta_{p},\;b_{i}\right)=\frac{e^{\left(\theta_{ps}-b_{i}\right)}}{1+e^{\left(\theta_{ps}-b_{i}\right)}},$$ (1)

where the subscript s represents subset s = 1, 2, …, S, *p* represents examinee, and *i* represents item. *θ*_*ps*_ is defined as the sum of primary traits among subsets and distinct traits by subset s. Specifically, let us consider a scale composed of s = 1, 2, …, S and let

$$\theta_{ps}=\theta_{p}+{\text{c}}_{\text{s}}\theta_{ps}^{\prime }$$ (2)

where *θ*_*p*_ is the primary trait among subsets, and $\theta_{ps}^{\prime }$ is the distinct trait characterized by subset s. It is hypothesized that *θ*_*p*_ is not correlated with $\theta_{ps}^{\prime }$ and neither are any $\theta_{ps}^{\prime }$ among any subsets mutually correlated. This indicates that $\theta_{ps}^{\prime }$ is a trait unique to each subset. The value of c_s_ is the magnitude of each specific subset s and c_s_ > 0 [19]. The correlation between the estimated traits of among any subsets s and t is given by $\rho_{st}=1/\sqrt{1+c_{s}}\sqrt{1+c_{t}}$. Assuming *c*_*s*_ = *c*_*t*_ = *c* gives *ρ*_*st*_ = 1/(1 + *c*^2^) (Marais & Andrich, 2008). By setting the magnitude of trait dependence 0, 1, and 2, the correlation between estimated traits among subsets is 1.0, 0.5, and 0.2, respectively. The correlation of 1.0 between traits among subsets indicates no violation of unidimensionality in the Rasch model. On the other hand, traits correlation among subsets less than 1.0 indicates violation of unidimensionality depending on the magnitude of trait dependence term, c.

#### Response dependence

Marais and Andrich [19] also suggested that RD can be considered as a violation of statistical independence. In other words, under RD, examinees’ response to an item depends on their response to a previous item. For example, if an examinee was able to give a correct answer for a previous item, he or she is more likely to get the next item correct. On the other hand, if the examinee gave an incorrect answer for the previous item, he or she is less likely to get the next item correct. This notion also generalizes to any set of binary, ordered response data (e.g., for an opinion-based item, 0 is disagree and 1 is agree). Under the Rasch model, RD can be formulized as following two equations:

$$P\left(X_{jp}=1|X_{ip}=1;\theta_{p},\;b_{i}\right)=\frac{e^{\left(\theta_{p}-\left(b_{j}-d\right)\right)}}{1+e^{\left(\theta_{p}-\left(b_{j}-d\right)\right)}},$$ (3)

and

$$P\left(X_{jp}=1|X_{ip}=0;\theta_{p},\;b_{i}\right)=\frac{e^{\left(\theta_{p}-\left(b_{j}+d\right)\right)}}{1+e^{\left(\theta_{p}-\left(b_{j}+d\right)\right)}},$$ (4)

where *p* represents the examinee, *i* represents the preceding item, and *j* represents the following item. This formula indicates that the response of an examinee to item *j* depends on the examinee’s response to item *i*. The value of d is adding or subtracting depending on whether the examinees’ response to preceding item was correct or endorsing. Specifically, if an examinee’s response to item *i* is correct or endorsing, the difficulty level of the following or dependent item *j*’ is made lower by the subtraction of the response dependence term (i.e., d). This makes the following or dependent item easier, which results in an increase in the examinee’s probability of getting the following or dependent item correct or endorsing. On the other hand, if an examinee’s response to item *i* is incorrect or not endorsing, the difficulty level of the following or dependent item *j*’ is made higher by the addition of the response dependence term (i.e., d). This makes the following or dependent item harder, which results in a decrease in the examinee’s probability of getting the following or dependent item correct or endorsing.

### Model fit indices for CFA with WLSMV estimation

In order to deal with non-normality with categorical indicators, WLSMV methods have been recommended over the ML method. WLSMV is a limited information estimation method that utilize summary statistics (i.e., tetrachoric or polychoric correlations). As a limited information method, WLSMV is not only a robust method but also computationally fast, especially when the sample size and the number of dimensions are large [20–23]. Thus, this study utilizes the WLSMV-based approach on CFI, TLI, and RMSEA indices. The calculations of those indices are presented below as Mplus, as is used in the study [24].

Both CFI and TLI come from the *χ*^2^ statistic obtained through the WLSMV estimation. They compare those statistics to the WLSMV *χ*^2^ statistic obtained from a baseline modeling. The baseline model collectively places zero to all item parameters in the categorical data except thresholds. After obtaining *χ*^2^ for both, the CFI and TLI are calculated as shown below.

$$CFI=1-\frac{\text{max}\left(\chi_{t}^{2}-df_{t},0\right)}{\text{max}\left(\chi_{t}^{2}-df_{t},\;\;\chi_{b}^{2}-df_{b},0\right)},$$ (5)

and,

$$TLI=\frac{\chi_{b}^{2}-\chi_{t}^{2}\left(\frac{df_{b}}{df_{t}}\right)}{\chi_{b}^{2}-df_{b}},$$ (6)

where *b* represents the baseline model, *t* represents the tested model, and *df* represents degrees of freedom.

The RMSEA [25] indices apply a fit statistic in quantifying the average discrepancies arising between expected and observed covariance within a specific data set in the defined latent variable model. The use of RMSEA in categorical data should be adjusted to account for the non-normality in the observed data. This is achieved by obtaining *χ*^2^ from the WLSMV estimation, which enables the calculation of RMSEA as

$$RMSEA=\sqrt{\frac{\chi^{2}-df}{df\left(N-1\right)}},$$ (7)

where N represents the sample size, and *df* represents the degree of freedom. The RMSEA takes into account the model degree of freedom and sample size. The degree of freedom is considered a measure of model complexity. The rationale is that for more degrees of freedom, there is an increase in the number of variable relations in the model. Similarly, the relation between sample size and degrees of freedom is incorporated into the RMSEA value, which is standardized.

In CFA practices, model fit indices are commonly used to gauge model misspecification. Since fit is a matter of degree, Hu and Bentler [26] have provided recommended cutoff criteria for those indices (e.g., CFI/TLI > 0.95, RMSEA < 0.06), which are now widely used in CFA applications. However, those are maximum likelihood based fit indices for continuous variables and less is known about WLSMV-based fit indices for ordered categorical variables [3]. Recent study [27] showed that it is not appropriate to use Hu and Bentler’s conventional cutoff benchmarks (i.e., CFI, TLI, RMSEA) for ordered categorical variables with WLSMV estimation. Instead of using fixed cutoff criteria, they suggested WLSMV based fit indices would be used as diagnostics tools for model specification.

### Principal component analysis on residuals in rasch model

The Rasch model assumes that the measurement model is unidimensional. The idea of PCAR is that there should be no meaningful pattern among the item residuals, after controlling for the single latent factor of the items by the Rasch model. The magnitude of the eigenvalue of the first component is considered to be an indicator of violation of unidimensionality. The rule of thumbs of the magnitude of the value depends on previous research. For example, Smith [17] insisted the value of higher than 1.5 implies a lack of unidimensionality under 500 persons and 30 items. Smith & Miao [16] suggested the value no less than 1.4 implies a lack of unidimensionality, while several studies [14, 18] suggested the value no less than 2.0 in assessing unidimensionality assumption. Although no fixed cut point would be applicable as Chou and Wang [28] pointed out, PCAR has been widely used to examine dimensionality in Rasch models by many practitioners.

## Methodology

### Design overview

A simulation study was conducted in R 4.0.2 [29], with batching of estimation to Mplus via Mplus Automation package [30]. For PCAR, the *eRm* package [31] and the psych package [32] was used. A summary of the simulation method is presented here. The following subsections provide details for each part of the method.

Factors manipulated in this study include type of LD (TD or RD), magnitude of LD, sample size, and number of items. Each simulation condition was replicated a thousand times. The item factor model constrains all loadings to be equal, which results in the same parameter estimation as the binary Rasch model. The Rasch model was fit to all data sets to estimate person and item parameters. Root mean square error (RMSE) were calculated as the evaluation criteria of parameter recovery. A series of correlational and graphic analyses was conducted to investigate the relationships between RMSE of parameter estimates and fit indices from WLSMV estimation.

### Data generation

Simulated data were generated from the Marais and Andrich’s Rasch model [19] for TD and RD, respectively (See Eqs 1 through 4). For the violation of local independence in Rasch model due to RD, the simulated data was generated by Eqs 3 and 4.

#### Simulation conditions

The basic design structures for both models are the same and consist of six subsets with dependence, TD or RD. All thetas including primary and subset specific thetas were drawn independently from standard normal distribution and item difficulties were drawn from -2 (i.e., easiest) to 2 (i.e., hardest) with equal intervals. The critical factor towards examining the research questions was varying the amount of LD in the generated data. The variation process on the amount of LD was varied for TD and RD. To align with Marais and Andrich (2008b), the constant c was varied from 0, 1, or 2 from the formula 1 for TD in the Rasch model; the constant d was varied from 0, 1, or 2 from the formula 3 and 4 for RD in the Rasch model. Under each manipulation of TD or RD, the number of items and sample size were varied in this study. To investigate the effect of test length factor, different number of items was considered. For smaller tests, the number of items was 30; to incorporate conditions associated with longer tests we also considered 60 items. The 30 items were distributed in six subsets of five items each, and the 60 items were distributed in twelve subsets of five items each. The first items of each subsets are the hardest ones as they are the preceding halo items that impact the following items of each subset. Based on the sample size condition from previous literatures [33, 34], 250, 500, and 1,000 sample sizes were considered in this study.

#### Data analysis and evaluation criteria

The simulated data was fit to the Rasch model [35]. The model calibrations were completed in Mplus with WLSMV estimation. The use of Mplus WLSMV estimations provided WLSMV-based fit indices such as CFI, TLI, and RMSEA in the Rasch model as earlier defined in Eqs 5 through 7.

After fitting the Rasch model and estimating ${\hat{\theta }}_{p}$, the RMSE index was calculated for $\hat{\theta }$ as shown below [36].

$$\hat{\theta }\;\text{RMSE}=\sqrt{\frac{\sum_{p=1}^{N}{\left({\hat{\theta }}_{p}-\theta_{True,p}\right)}^{2}}{N-1}},$$ (8)

where ${\hat{\theta }}_{p}$ is the estimated ability for examinee p, *θ*_*True*,*p*_ is the known true ability value for examinee p, and N is the total number of examinees.

Analogous RMSE values for $\hat{b}$ was calculated for each iteration of the simulation.

$$\hat{b}\;\text{RMSE}=\sqrt{\frac{\sum_{i=1}^{I}{\left({\hat{b}}_{i}-b_{True,i}\right)}^{2}}{I-1}},$$ (9)

where ${\hat{b}}_{i}$ is the estimated difficulty for item i, *b*_*True*,*i*_ is the true item difficulty parameter for item i, and I is the total number of items.

## Results

Results are presented in three sections. Results from TD and RD are presented for the relationship between WLSMV-based fit indices (i.e., CFI, TLI, RMSEA) and RMSE of parameter estimates (i.e., ability and item difficulty), and comparison between those indices and PCAR, respectively.

### Trait dependence

If the fit indices were helpful in detecting LD that results in RMSE in parameter estimates, there should be a relationship between WLSMV-based fit indices and RMSE of parameter estimates. Correlation analyses between those fit indices and such RMSE in the Rasch model are applied and results are presented in Table 1. Table 1 shows that both RMSE of ability and difficulty estimates are strongly correlated with all the fit indices in the expected direction (i.e., fit indices worsen as RMSE increases). These results imply that WLSMV-based fit indices are potentially useful to detect LD cause inaccurate estimates in ability and difficulty parameter in the Rasch model when trait dependence exists.

**Table 1:** Correlations between WLSMV-based fit indices and RMSE of parameter estimates.

TD					RD				
	RMSE*θ*	p-value	RMSE b	p-value		RMSE*θ*	p-value	RMSE b	p-value
CFI	-.87 [-87, -87]	< .001	-.83 [-83, -83]	< .001	CFI	-.26 [-26, -26]	< .001	-.46 [-46, -46]	< .001
TLI	-.87 [-87, -87]	< .001	-.83 [-83, -83]	< .001	TLI	-.25 [-25, -25]	< .001	-.46 [-46, -46]	< .001
RMSEA	.95 [.95, .95]	< .001	.88 [.88, .88]	< .001	RMSEA	.24 [.24, .24]	< .001	.29 [.29, .29]	< .001

*Note*. Values in square brackets indicate the 95% confidence interval for each correlation.

However, it is also shown that the relationships of each fit indices to RMSE of ability and difficulty estimates are dependent on simulation factors in this study (i.e., magnitude of trait dependence, number of items and sample size). The following sections will present the details.

#### Relationship between CFI/TLI and RMSE of ability and difficulty estimates with test factors

**Fig 1:**
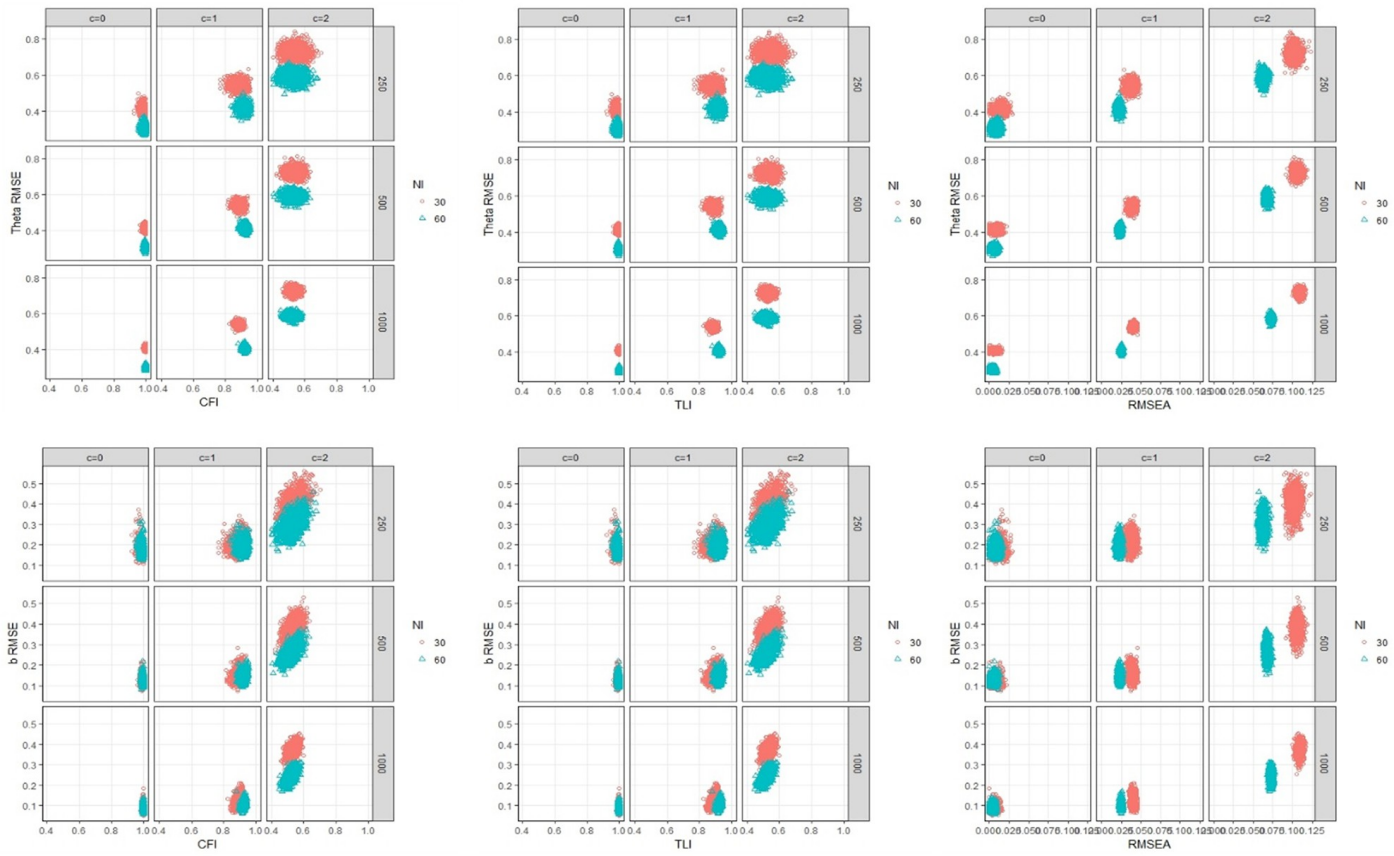
The relationship between WLSMV-based fit indices, theta RMSE/ $\hat{b}$ RMSE, and test factors (TD).

Since the correlation between CFI and TLI is very strong (r = 0.99), the results of those two fit indices are presented in the same section. The relationships between CFI/TLI and $\hat{\theta }$ RMSE are impacted by the interaction of two simulation factors—magnitude of trait dependence and number of items. As seen in Fig 1, the fit indices worsen as the magnitude of TD increases, which means those indices work in detecting the presence of local dependence due to TD. Specifically, both CFI and TLI are mainly impacted by the magnitude of trait dependence. Notice that local independence assumption is met when c = 0 that shows good fit results above 0.95 even sample size is 250 in both CFI and TLI. When the trait dependence exists (i.e., c = 1 or c = 2), almost all fits are less than 0.95 but only 0.8% cases shows over 0.95 when c = 1 and no cases over 0.95 when c = 2. Test length is another factor that influences $\hat{\theta }$ RMSE but does not relate to CFI/TLI. More test items produce less $\hat{\theta }$ RMSE, and yet the length of the test is not related to CFI/TLI within all sample size condition. This result indicates that $\hat{\theta }$ RMSE results are more sensitive to number of items than are CFI/TLI fit results.

For the relationships between CFI/TLI and $\hat{b}$ RMSE, when the local independence assumption is met (i.e., c = 0), both test sample size and test length are not factors that impact the relationship between CFI/TLI and $\hat{b}$ RMSE, and in such instances an acceptable CFI/TLI is aligned with low $\hat{b}$ RMSE. When a moderate magnitude of trait dependence exists (i.e., c = 1), number of items impacts the range of the resultant fit indices, but still only 0.8% cases shows over 0.95 (e.g., indicate less than good fit according to CFI/TLI). When a large magnitude of trait dependence exists (i.e., c = 2), there is a positive linear relationship between CFI/TLI and $\hat{b}$ RMSE, and more test items produce less $\hat{b}$ RMSE while having no particular influence on CFI/TLI.

#### Relationship between RMSEA and RMSE of ability and difficulty estimates with test factors

The relationships between RMSEA and $\hat{\theta }$ RMSE are impacted by the interaction of two simulation factors—magnitude of trait dependence and number of items. Considering the property of RMSEA that the smaller RMSEA the better fit, RMSEA works (i.e., increases) when the magnitude of trait dependence is increased. However, RMSEA is impacted not only by magnitude of trait dependence but also by number of items, unlike CFI/TLI. This is true for $\hat{b}$ RMSE as well presented in Fig 1. Specifically, there are no major differences between RMSEA values across the simulation iterations and conditions in which the trait local independence assumption is met (i.e., c = 0); but when the trait local independence assumption is violated (i.e., c = 1 or c = 2), test length is a factor that influences both $\hat{\theta }$ /$\hat{b}$ RMSE and RMSEA. More test items produce less $\hat{\theta }$ /$\hat{b}$ RMSE and RMSEA values within all sample size condition.

### Response dependence

For the response dependence scenario, the correlation results show a less strong relationship between fit indices and RMSE of parameter estimates as compared to the trait dependence scenario. Table 1 shows that both RMSE of ability and difficulty estimates are small to moderately correlated with the fit indices though all the fit indices are significant and show expected directions.

#### Relationship between CFI/TLI and RMSE of ability and difficulty estimates with test factors

**Fig 2:**
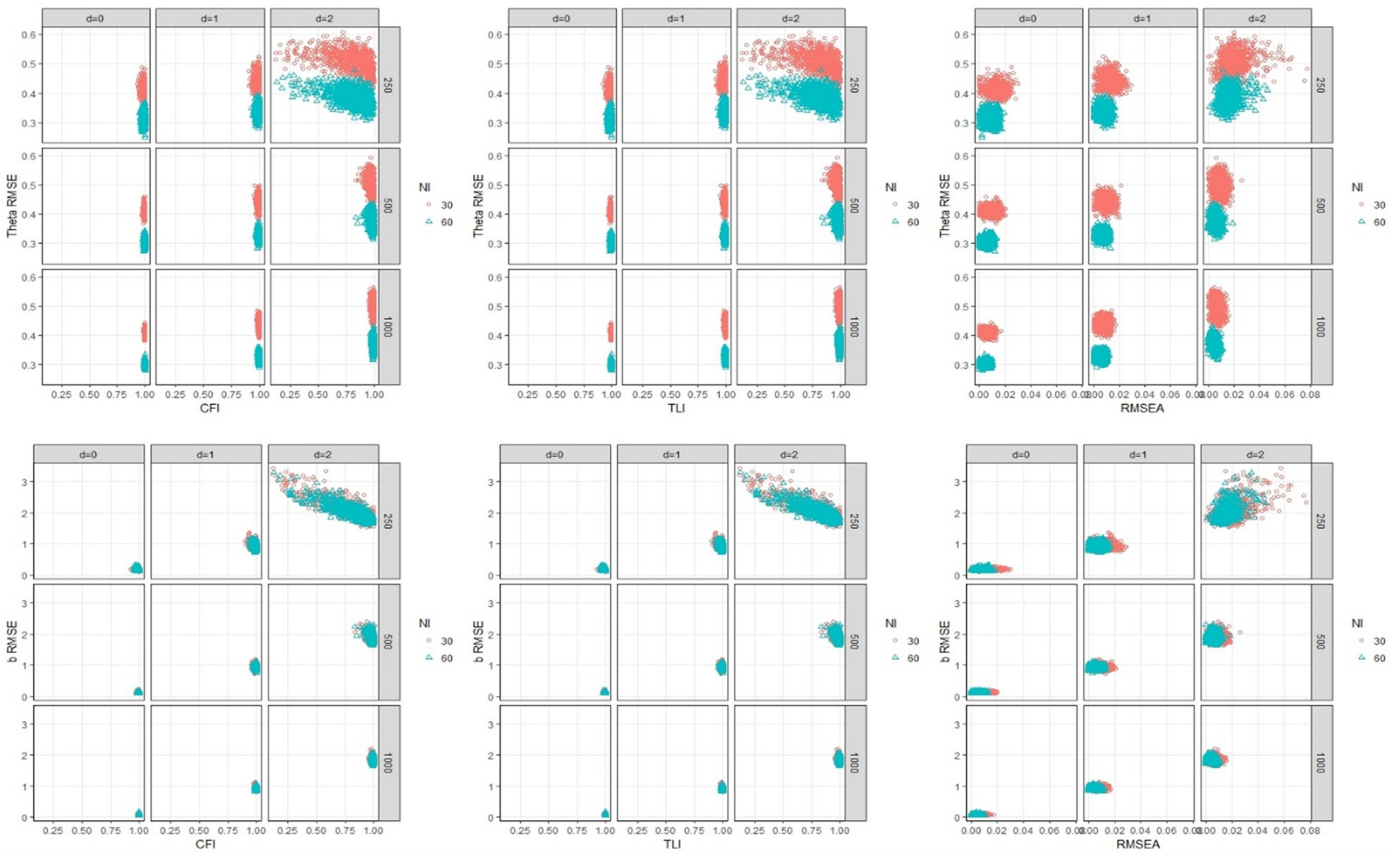
The relationship between WLSMV-based fit indices, theta RMSE/$\hat{b}$ RMSE, and test factors (RD) relationship between RMSEA and RMSE of ability and difficulty estimates with test factors.

In Fig 2 both CFI/TLI demonstrate that the relationships between CFI/TLI and $\hat{\theta }$ RMSE are mainly impacted by number of items. In all RD simulation conditions, CFI/TLI results produce perfect fit except one condition (i.e., d = 2 & sample size = 250), which indicates that CFI/TLI seem not to work in detecting the presence of local dependence due to RD. Regarding parameter RMSE estimates, test length impacts $\hat{\theta }$ RMSE but not on $\hat{b}$ RMSE. Neither number of items nor sample size has an impact on the relationship between CFI/TLI and $\hat{b}$ RMSE, as can be seen in Fig 2. $\hat{b}$ RMSE is only affected by the magnitude of response dependence and $\hat{\theta }$ RMSE is only affected by number of items when response dependence violation occurs.

The relationships between RMSEA and $\hat{\theta }$ RMSE are impacted by sample size and the interaction of that effect with RD magnitude. In Fig 2, RMSEA seem not to work in detecting the presence of local dependence due to RD as with CFI/TLI cases. Regarding parameter RMSE estimates, test length only impacts $\hat{\theta }$ RMSE but not $\hat{b}$ RMSE. Neither number of items nor sample size has an impact on the relationship between RMSEA and $\hat{b}$ RMSE, as can be seen in Fig 2. $\hat{b}$ RMSE is only affected by the magnitude of response dependence and $\hat{\theta }$ RMSE is only affected by number of items when response dependence violation occurs.

### Comparison of PCAR and WLSMV fit indices

In each simulation condition, PCAR was conducted to produce the eigenvalue of the first component. As those values are used to determine whether the data are unidimensional in the Rasch model, it would be beneficial to compare those to fit indices from WLSMV estimation. It is noted that PCAR is a method for checking unidimensionality that corresponds to local dependence due to TD in this study. As noted in response dependence section above, the fit indices from RD simulation condition produce nearly perfect fit regardless of magnitude of RD. Therefore, the result of comparison is presented mainly about TD with regards to the first eigenvalue of PCAR.

**Fig 3:**
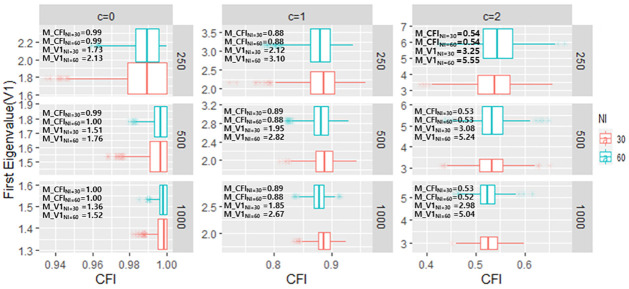
The relationship between first eigenvalue of PCAR and CFI in each TD simulation conditions. (*Note*. M: mean of values across simulations).

**Fig 4:**
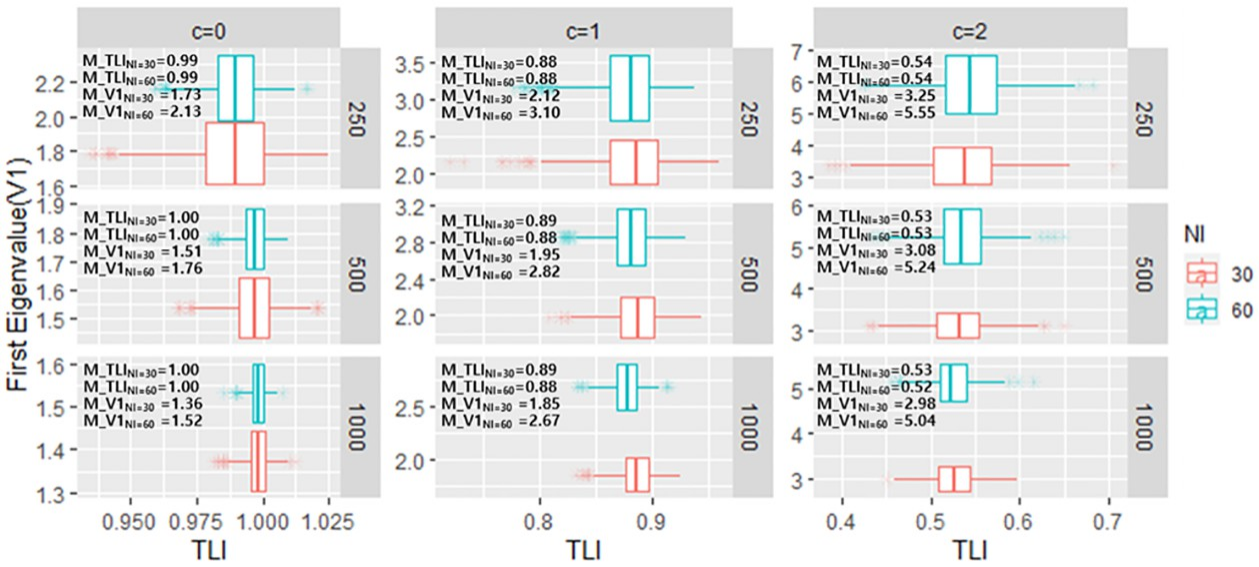
The relationship between first eigenvalue of PCAR and TLI in each TD simulation conditions. (*Note*. M: mean of values across simulations).

**Fig 5:**
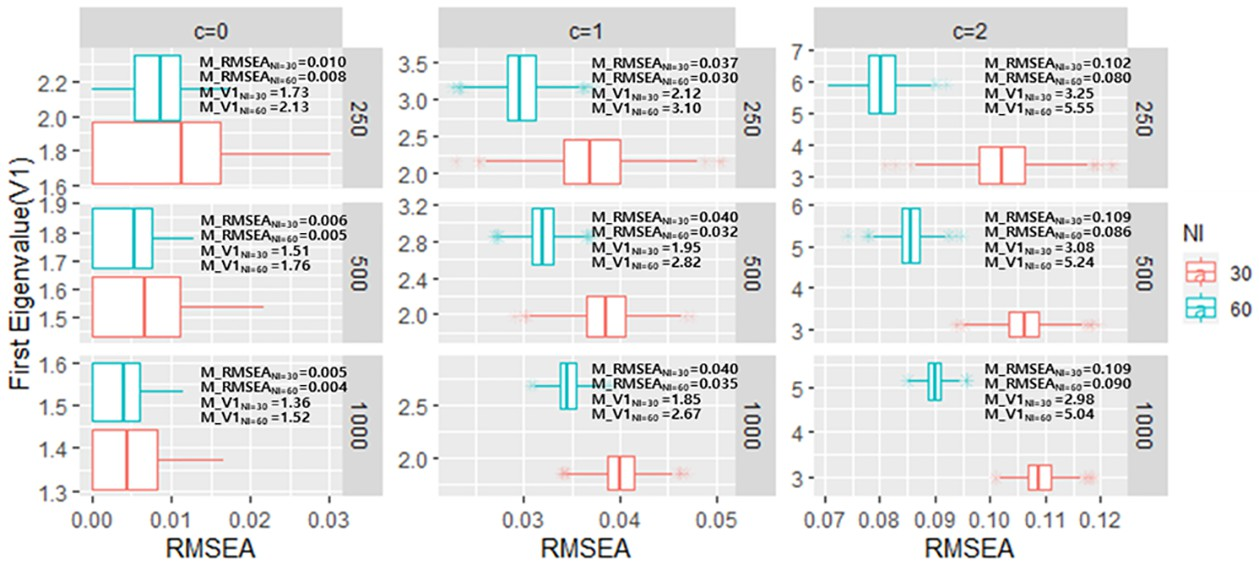
The relationship between first eigenvalue of PCAR and RMSEA in each TD simulation conditions. (*Note*. M: mean of values across simulations).

The relationship between CFI/TLI/RMSEA and first eigenvalue of PCAR (V1), and mean of those values are presented in Figs 3–5, respectively. Within each sample size conditions, when c increases (i.e., the magnitude of TD increases) both CFI/TLI/RMSEA and V1 worsens. That is, it is much expected for those values to be varied as TD occurs. However, considering the test length factor, those values produce different scenarios. Specifically, CFI/TLI/RMSEA is very consistent within each test length condition (e.g., mean of CFI in c = 1 & sample of 250 is equal to 0.88 in both 30-item and 60-item condition), whereas V1 depends on test length condition (e.g., mean of V1 in c = 1 & sample of 250 is equal to 2.12 in 30-item and 3.10 in 60-item, respectively). The results align with Chou and Wang [28]’s simulation study as they pointed out that V1 depends on sample size and test length.

## Discussion

This study utilizes Marais & Andrich’s [19] violation of local independence assumption in the Rasch model for simulating data, and then applies binary Rasch models to estimate item and person parameters. The simulation allowed for a comparison of conditions that vary in magnitude of trait and response dependence violations, as well as other factors including sample size and number of test items.

The idea stemmed from Huggins-Manley and Han’s [4] previous research about 2-PL IRT models. They utilized multidimensional IRT models to simulate LD that counterparts to TD in this study; therefore, it is worthwhile to compare the results from TD to the results from their previous study. Huggins-Manley and Han’s [4] found that test factors (i.e., number of items & sample size) were strongly related to RMSE in ability and difficulty parameters, respectively. However, the relationship between the fit indices and RMSE in item discrimination parameters was not as clear as the relationship between fit indices and RMSE in ability and difficulty parameters. For discrimination parameter, the relationship was associated with sample size as well as correlation among traits that is hard to control a priori in practical settings. Due to the complexity of discrimination parameters, it was expected that the Rasch model produces a more explicit relationship between fit WLSMV-based fit indices and LD-induced inaccurate parameter estimates. This study supported this claim for TD, but not for RD. The results of TD indicate that the WLSMV-based fit indices and RMSE of parameter estimates are closely related to each other. When TD occurs, the fit indices might be able to detect the violation because fits worsen as the magnitude of TD increases.

It is worth noting that there are many instances in which either the fit indices or the parameter estimate RMSE is influenced by a factor (sample size or number of items), but the factor does not influence both. For example, it was shown that when TD increases to a large magnitude, RMSE of person ability and item difficulty also increase, and the increase is larger for shorter tests as compared to longer tests. However, the CFI/TLI values were the same across the different test lengths. Therefore while CFI/TLI can flag TD forms of LD, they were not sensitive to the same factors that resulted in LD-induced RMSE in person and item parameter estimates. This is one of many indications that the fit indices are useful for flagging LD concerns, but that any cutoff value used for CFI/TLI would not take into account the fact that LD-induced RMSE in parameter estimates is dependent on sample size. This finding aligns with Huggins-Manley and Han’s (2017) findings.

On the other hand, fit indices are not able to detect the RD violations because all the fit results are constant as the magnitude of RD increases. Within the RD situation, the response patterns were highly distorted in cases where the true ability and item difficulty were exactly the same. This is because the responses of the rest of the items were dependent only on the response of the first item rather than the true ability and item difficulty. This does not impact on the global fit indices despite the distorted pattern due to RD. The estimated parameter with the discrepancy from the true parameter might show good fit statistics under the occurrence of RD.

Is it useful to use WLSMV-based global fit indices in detecting LD for Rasch applications? The answer would be "yes" especially in the situation of TD occurs. Based on the results from the comparison of PCAR, WLSMV-based global fit indices have meaningful advantages over the use of V1. Both WLSMV-based global fit indices and V1 are expected to be varied as the magnitude of TD increases, however, WLSMV-based global fit indices are consistent regardless of sample size and test length while V1 is not. This result implies that the decision based on a fixed cut point of V1 with regard to assessing unidimensionality assumption in the Rasch models would not be appropriate, indicated by Chou and Wang [28] as well. Regarding WLSMV-based global fit indices, those would possibly be used for the detection of TD. As Xia and Yang [27] pointed out, however, that it is not appropriate to set cutoff values for WLSMV-based fit indices from ordered categorical data. Though this study does not aim to address the question of what the new cutoff values from WLSMV-based fit indices should be employed, we would know those indices are related to the violation of TD, which do not depend on sample size and test length. For example, a researcher fits the Rasch model that results in good fit results (e.g., CFI>0.98, TLI>0.98, RMSEA<0.01), which indicates—at least—no violation of TD. However, given the relationships between fit indices and misspecifications due to RD, it is not feasible to detect RD using WLSMV-based global fit indices since those fits are always good regardless of RD.

In educational or psychological setting, multidimensionality might occur in an assessment that results in LD [37]. This type of violation of local independence assumption is defined as TD [19]. Based on the findings, WLSMV-based global fits can be useful in detecting TD for the educational or psychological assessment types. In contrast, it is often found that RD occurs in health or physical-related field of research for the application of Rasch rating scale. On a rating scale measuring physical functioning, RD can be found when a subset of items have some features in common [38]. For instance, measuring walking ability questions may have RD due to similarities in response format or item content [39, 40]. Marais [41] presented several examples of RD occurrence, which is caused by types of questions (e.g., asking overall level of status following several other status asking items or an item is made negative of the other preceding item). Marais [41] also pointed out that RD can be found where raters make judgements using an instrument consisting of a number of criteria (i.e., “halo effect” followed by different raters if they judge similar ratings on the different criteria than they would be if rated independently). Therefore, it should be noted that using WLSMV-based global fit indices might not be useful under the circumstances of application of those kinds of rating scales especially to have RD.

It turns out that WLSMV-based global fit indices are much more sensitive to TD than RD, even though both are a form of LD. Therefore, focusing on Research Question 2 and test factors (i.e., number of items and sample size) in TD situation, both CFI and TLI are not impacted by test factors but only by trait dependence violation. But RMSEA is impacted by number of items especially when TD occurs that more items produce better RMSEA fit though the same magnitude of trait dependence is violated. This is an indication that parameter estimates in Rasch models become more robust to TD violations of local independence as the number of items increases. Also, this is an indication that cutoff scores should not be applied to the WLSMV-based global fit indices when fitting Rasch models because the appropriate cutoffs would vary by number of items.

Based on all of the findings, some summarizing statements are provided here. First, WLSMV fit indices of CFI, TLI, and RMSEA are not sensitive to violations of local independence due to RD, and their use is not recommended for detecting RD. Second, WLSMV fit indices of CFI, TLI, and RMSEA are quite sensitive to violations of local independence due to TD. The larger the TD effect, the higher the RMSEA value becomes. The larger the TD effect, the lower the CFI and TLI value becomes. However, the magnitude of RMSE of parameter estimates that is associated with particular values of WLSMV-based CFI, TLI and RMSEA is dependent on number of items, cutoff values for these fit indices are not recommended. Generally speaking, longer tests are associated with lower RMSE of parameter estimates while length of test does not impact the magnitude of WLSMV CFI, TLI, and RMSEA.

Finally, several limitations are addressed in this study. First, this study only considers dichotomous responses, but future research should include polytomous responses. Second, further investigation or justification are needed about the magnitude of TD or RD (i.e., c or d) that are varied as integers like 0, 1, 2 in this study. The c term is associated with the correlation between estimated traits among subsets (see footnote 1 on page 5), while the d term is just integer values adding or subtracting from item locations in the formula. It might be interesting to consider different levels of RD (e.g., d = 1.5), as is also the case with TD. Third, regarding the test design, this study applied a subtest design with a fixed number of items, but it is unknown whether the results will be the same with a different number of subtests or no subtest design. Also, sixty items, as well as one thousand examinees, may be considered quite large for some applications of Rasch in practice, such that future research may want to consider a fewer number of items or examinees for practical purposes. Lastly, this study focuses on assessing LD, especially global fit of the Rasch model using WLSMV-based fit indices. In this controlled study, when these WLSMV-based fit indices indicated misfit, this was due to TD, as TD was the source of misfit that was being manipulated. However, misfitting WLSMV-based fit indices will not always be due to TD as there are many other aspects to consider (e.g. individual item-fit, person fit, differential item functioning, targeting, etc.). As with any global fit indicator, the WLSMV-based fit indices will indicate an issue within the item set, but it will not reveal the nature of the issue, and therefore the item set would still require further investigation.

To conclude, final answer statements with regard to research questions addressed in this study. Research question 1 asked CFI, TLI, and RMSEA indices from WLSMV estimation of binary Rasch models were sensitive to LD-induced bias in item and person parameter estimates. For each parameter estimates, bias from LD had significant correlated relationships to each fit indices in expected directions. It should be noted that the relationship between fit indices and parameter estimates are much strong in TD simulated condition. Research question 2 asked if the answer to question 1 was dependent on various simulated factors. For person parameters, the relationship between the fit indices and bias in person parameter estimates was affected by number of items. For item parameters, the relationship between the fit indices and bias in item parameter estimates was affected by the sample size. Research question 3 asked if the WLSMV-based fit indices useful in the comparison of PCAR. The fit indices were robust to various studied factors as the magnitude of LD (especially for TD) increases while the result from PCAR was not. The results reveal the usefulness of the method proposed in this study over the use of PCAR.
